# Effects of task and age on the magnitude and structure of force fluctuations: insights into underlying neuro-behavioral processes

**DOI:** 10.1186/s12868-015-0153-7

**Published:** 2015-03-13

**Authors:** Solveig Vieluf, Jean-Jacques Temprado, Eric Berton, Viktor K Jirsa, Rita Sleimen-Malkoun

**Affiliations:** Aix-Marseille Université, CNRS, Institut des Sciences du Mouvement UMR 7287, 13288 Marseille cedex 09, France; Aix-Marseille Université, Inserm, Institut de Neurosciences des Systèmes UMR_S 1106, 13385 Marseille, France

**Keywords:** Aging, Variability, Signal-to-noise ratio, Complexity, Multi-scale entropy, Frequency analysis

## Abstract

**Background:**

The present study aimed at characterizing the effects of increasing (relative) force level and aging on isometric force control. To achieve this objective and to infer changes in the underlying control mechanisms, measures of information transmission, as well as magnitude and time-frequency structure of behavioral variability were applied to force-time-series.

**Results:**

Older adults were found to be weaker, more variable, and less efficient than young participants. As a function of force level, efficiency followed an inverted-U shape in both groups, suggesting a similar organization of the force control system. The time-frequency structure of force output fluctuations was only significantly affected by task conditions. Specifically, a narrower spectral distribution with more long-range correlations and an inverted-U pattern of complexity changes were observed with increasing force level. Although not significant older participants displayed on average a less complex behavior for low and intermediate force levels. The changes in force signal’s regularity presented a strong dependence on time-scales, which significantly interacted with age and condition. An inverted-U profile was only observed for the time-scale relevant to the sensorimotor control process. However, in both groups the peak was not aligned with the optimum of efficiency.

**Conclusion:**

Our results support the view that behavioral variability, in terms of magnitude and structure, has a functional meaning and affords non-invasive markers of the adaptations of the sensorimotor control system to various constraints. The measures of efficiency and variability ought to be considered as complementary since they convey specific information on the organization of control processes. The reported weak age effect on variability and complexity measures suggests that the behavioral expression of the loss of complexity hypothesis is not as straightforward as conventionally admitted. However, group differences did not completely vanish, which suggests that age differences can be more or less apparent depending on task properties and whether difficulty is scaled in relative or absolute terms.

## Background

### Variability: a marker of neuro-behavioral functioning

When a person is performing a motor task, behavioral output is characterized by fluctuations over time. Behavioral variability is also a generic manifestation of aging in the neuro-musculo-skeletal system (NMSS). Indeed, it has been repeatedly observed that aging leads to a significant increase in intra-individual variability of cognitive and motor outputs [1,2]. According to information theory and the neural noise hypothesis, such increase in variability might lead to lower information processing efficiency [3-5], that is currently measured by the signal-to-noise ratio (see [6] for an example in Fitts’ task).

However, the increase in the magnitude of behavioral variability is not the only consequence of aging. Indeed, its dynamic structure, i.e., deterministic rules and correlated fluctuations, can be also affected. Specifically, it is widely recognized that the *loss of complexity* of behavioral output fluctuations – i.e., change in time-structure of variability toward either an increase or a decrease in the regularity measured by entropy metrics (e.g., approximate entropy, ApEn) [7-10] – is a functional indicator of organismic functions in health and disease. It may as well contribute to the better understanding of age-related reorganization of physiological and motor control systems [10-14].

The distinction between the amplitude and the structure of variability is based on the assumptions that: (i) random/uncorrelated variability extracted from the signal’s variance is a proxy of the amount of white Gaussian (neural) noise in the system and an index of its efficiency through the measure of signal-to-noise ratio (e.g., [15]); and (ii) the structure of variability, which is reflected in the signal’s complexity, is a proxy of the underlying organization of the multiple components or processes involved at different temporal and spatial scales in task performance [9,13,15]. Presumably, the magnitude of variability and its time-correlation structure stem from different origins and, accordingly, have specific functional significance. However, the question arises as to how the amplitude of variability (and, hence, the signal-to-noise ratio) and its time-structure evolve in principled ways as a function of task constraints (see [16] for an illustrative example in bimanual coordination) and during aging. One of the objectives of the present study was to address this issue in an isometric force control task.

### Isometric force production: a prominent paradigm in motor control

Isometric force control is one of the most prominent paradigms that are currently used in motor control and aging research to explore the different mechanisms underlying perceptuo-motor variability [17-23]. It has been demonstrated that force output fluctuations around a target value are sensitive to task demands, e.g., the required force level. Further, variability of the force output also changes with age [15,19,24-27]. This latter effect is often attributed to age-related changes in motor unit recruitment and/or firing rate to maintain a given level of force output [28,29]. However, age-related changes in force control are not limited to the product of intra-muscular alterations. They are actually also driven by task-specific control and coordination constraints [19]. Indeed, as in many functional tasks, accurate control of magnitude, direction, and timing of force results from a coalition of multiple constraints of various origins (neural, cognitive, neuro-muscular, musculo-tendinous, energetic, etc.) and, in particular, from central integration of different sensory feedback loops with specific time delays [10,30,31]. The involvement of different control processes in task-goal achievement can be assessed through the relative amount of power expressed in specific frequency ranges (for an overview see [32]). From this perspective, low frequency bands (0–4 Hz) are associated with sensorimotor processing [33-35], whereas higher frequency bands (8–12 Hz) are considered to reflect the neural components of physiological tremor [36]. It has been suggested that with increasing difficulty of the task, the relative contribution of low frequency displays an inverted-U shape evolution with a minimum around 40% of MVC [15,34]. Compared to young adults, elderly were found to present greater relative power in the 0–4 Hz bandwidth [23,37], which was presumably associated with deficits in visuo-motor processing capacities (e.g., [38,39]).

Overall, changes in magnitude, structure, and frequency content of force fluctuations convey valuable information about underlying modifications and reorganizations between the aforementioned control mechanisms as a function of force levels and age. In the present study, we hypothesized that these changes should occur concomitantly in principled ways, as a signature of (self-organizing) dynamics between functional mechanisms underlying force- and age-related changes in efficiency and complexity of the neuro-behavioral system [15,28].

### Variability, information transmission, and complexity in isometric force control

In commonly used force control tasks where subjects are requested to either maintain or modulate their forces as instructed by a visually presented target line (constant or variable force over time), the magnitude of behavioral fluctuations is measured in either absolute terms (i.e., via the standard deviation, SD) or in relative terms (e.g., via the coefficient of variation, CV, [19]). It has been shown that in young adults, SD increases non-linearly with increasing force level [15,28], whereas the CV is highest for low force levels [20,19]. Older adults are generally found to exhibit larger variability than young adults when expressed in relative terms [18,20,23,39]. The results for variability expressed in absolute terms are less consistent and vary between studies, from a significant [40] to a nearly significant (e.g., [39]) age effect or even no difference (e.g., [18]). It is noticeable, however, that the effect of age on these two metrics has not been systematically investigated across the range of possible (relative) force levels. Available findings regarding changes in the time-structure of force production with advancing age indicate that, at least for low force levels, the variability of force maintenance gets more regular, and thus less complex (i.e., ApEn decreases) [37,41].

Slifkin and Newell [15,34] addressed the issue of the co-variation between signal-to-noise ratio (relying on behavioral variance) and entropy measure during isometric force control in young adults. They assumed that maintaining a given level of isometric force results from interactions between multiple component processes (e.g., feedback loops, motor unit recruitment, firing rate of neural commands, or attentional control) [10]. According to this assumption they hypothesized that optimal complexity of the system’s organization and information processing efficiency should be functionally related. The different experiments carried out in both studies comprised force levels between 3 and 95% of the individuals’ MVC. Although a smooth pattern could be less consistently drawn from the second study, authors concluded that: (i) signal-to-noise ratio (efficiency function) and complexity measure (ApEn) followed a similar nonlinear trend (inverted-U curve) and (ii) the optima of the two curves roughly corresponded to the same range of force level (35–40%). These findings are compatible with the hypothesis that complexity of behavioral outputs is related to variability and information processing in the system [22,38]. In other words, the optimal organization of the NMSS, which is reflected in entropy metrics, presumably improves the efficient transfer and circulation of information necessary to produce and control force over time [42]. Such integrated view, which constitutes a theoretical alternative to the classic approach to neuro-muscular control, has not been completely elucidated and suffers from some limitations that preclude its extension to aging without further empirical investigation. To our knowledge, no other published work attempted to reproduce Slifkin and Newell’s [15] results.

### Variability of force output and the system’s efficiency: limitations and challenges

Beyond the above-mentioned inconsistencies in Slifkin and Newell’s study (cf., results of experiments 1, 2, and 3, Figure two in [34]), some other limitations deserve to be pointed out and addressed before (i) confirming the link between information processing in the system and the expressed complexity in behavioral outputs, and (ii) understanding how this link is affected by task and age factors. A major point is the single scale metric of entropy that was used (i.e., ApEn) to infer complexity of force fluctuations. Indeed, ApEn is known to be highly sensitive to data length and to produce less consistent and reliable results than its subsequent version, i.e., sample entropy (SampEn, [43,44]). Additionally, both single scale measures do not make the distinction between a completely random signal and a complex one that contains long-range correlations. For instance, white noise (random signal) always yields higher entropy values than pink noise (complex signal). To overcome this limitation and to offer a better differentiation between random and complex processes, Costa et al. [45] introduced multi-scale entropy (MSE) and showed that this measure is more reliable than the single scale estimators to characterize physiologic and sensorimotor complexity by taking long-term correlations into account (see [11,46] for detailed explanations).

Through the use of MSE, the different time-scales can be, to some extent, linked to different processes operating at certain bandwidths. This has been recently exploited in brain studies (see [47]; Sleimen-Malkoun et al., revised) but, to our knowledge, never in behavioral studies and, especially not, in force control experiments. The underlying reasoning is that the coarse graining procedure acts in similar fashion as a low-pass filter, which would result in eliminating progressively the processes operating at the fastest scales. Thus, MSE offers a valuable addition to the spectral slope (log frequency-log power plot) analysis that is more conventionally used to hint at changes in auto-correlations characterizing the signal, and thereby revealing information about underlying control processes (e.g.,[2]). For instance, Sosnoff and Newell [2] proposed that with age a more broadband profile of the frequency structure should be related to less structure in the variability, and could be hence indicative of a change in the number of active degrees of freedom. Using MSE, this should be reflected in time-scale dependent changes, thereby indexing the specific contribution of the different processes to the observed changes in complexity with increasing age.

### Aims and hypotheses of the study

In the present study, we aimed to attain a better understanding of what distinguishes between different conditions of functioning of the same system (i.e., different force levels) and different functional systems (i.e., young and older adults) during isometric force production. To achieve this objective, we analyzed complementary variables that characterize the changes in the amplitude and the structure of the variability of the force output. We hypothesized that a more global picture of task- and age-related differences could be afforded by linking them with mechanisms known to operate on specific time-scales and frequency bands. Accordingly, the comparisons with previous studies that used single scale entropy measures and/or different acquisition frequencies and filtering were made more straightforward by converting scale factor (number of iteration of the coarse graining) to time-scales (in ms).

For absolute variability as well as for frequency-based measures, we expected a non-linear increase with increasing force level. For the efficiency, we expected to observe an inverted-U shape function with a maximum around 35–40% of the MVC. In line with findings by Slifkin and Newell [15,34], we expected that global complexity measures of force fluctuations would match the efficiency functions, with roughly aligned optima at intermediate force levels. It was hypothesized that this matching would be more pronounced for the time-scales containing mostly information about lower frequencies that are relevant to sensorimotor processing (i.e., < 4Hz). Indeed, focusing on specific scales would inform about process-dependent changes in the signal’s complexity (when a range of scales is examined) and predictability (when one scale is considered).

With respect to aging effects, we expected older participants to be overall more variable, less efficient, and less complex in their force output. With regard to the age-by-force levels interaction, we tested two contrasting hypotheses: (i) the general organization and operating mechanisms would remain the same as the system ages but efficiency would be lower, inducing hence a downward shift in the values of the different measures regardless of the force level; or (ii) the underlying organization and control would breakdown at specific levels of constraints (i.e., force).

## Methods

### Participants

Eleven healthy older adults (mean age: 67 years; SD: 1.7; range: 62–79; 6 women) and eleven healthy young adults (mean age: 23 years; SD: 2.2; range: 20–28; 5 women) participated in this study. All participants took part voluntarily and provided their informed consent to the procedure of the study. They were not aware of the specific purpose of the study and it was stated that they could stop the experiment at any time they would wish to. Participants self-declared that they were healthy, physically active, and autonomous in daily living activities. Further, all participants reported to be right-hand dominant, to have normal or corrected-to-normal vision, and no trauma of upper limb or known disease that might affect the results of the experiment. The experimental protocol was approved by the local ethic committee of Aix-Marseille University and was in accordance with the ethical standards laid down in the Declaration of Helsinki.

### Experimental setup

**Figure 1:**
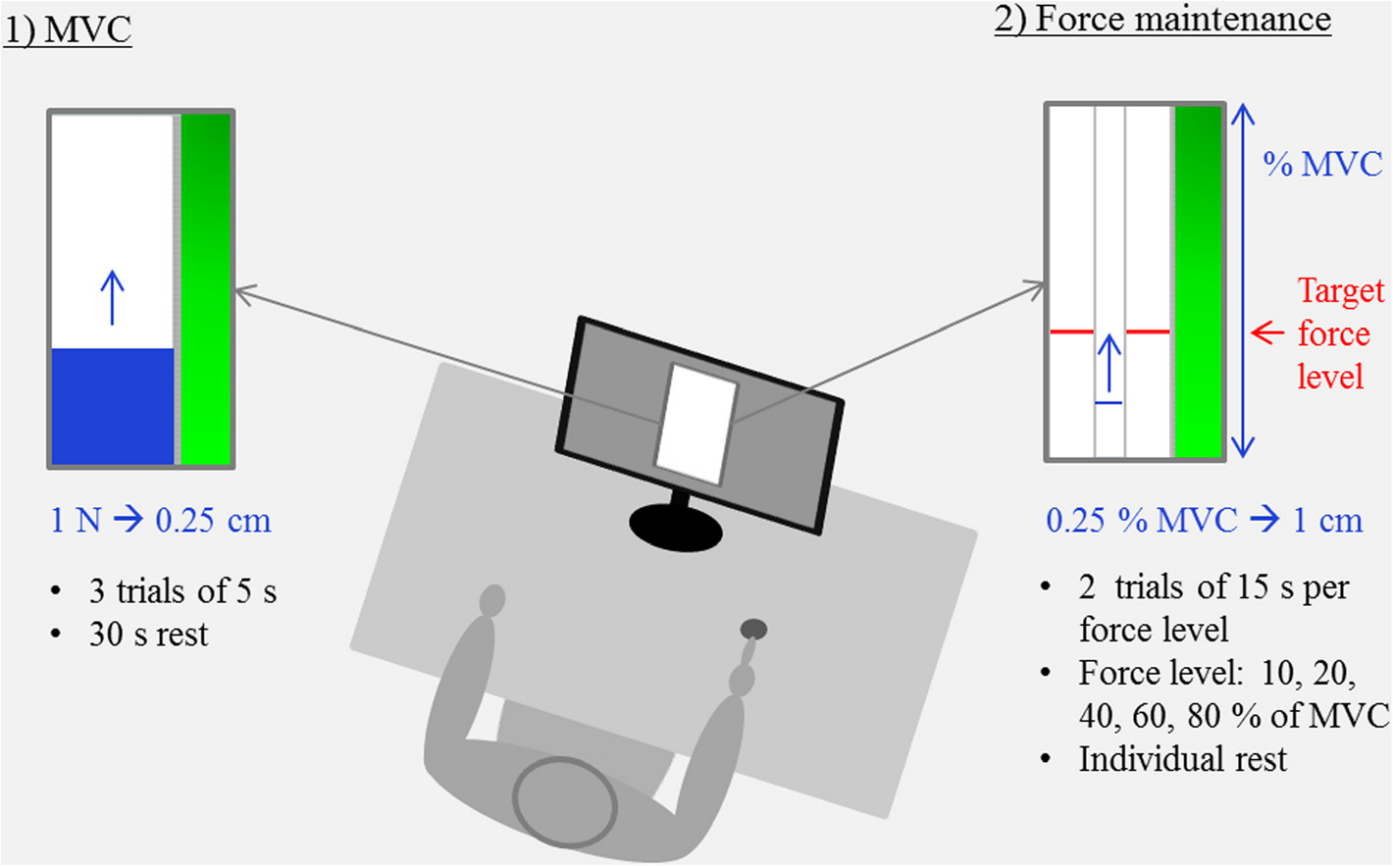
**Experimental setup.** Participants were seated in a comfortable position with both forearms resting on the tabletop so that the right index finger was easily placed on the force transducer. Visual feedback of their performance was provided on a screen in front of them. The experiment included two steps with specific feedback displays: 1) the MVC estimation (left) and 2) the force maintenance task (right). For the MVC, the pressure of the finger that was applied to the force transducer raised up a blue column by 0.25 cm per 1 N. For the maintenance task, two lateral red bars represented the target line (i.e., the force level in % MVC to reach) and a mobile blue bar restituted the applied force level.

Participants sat at an experimental table with their forearms resting on the table (see Figure 1), so that the right index finger was lying comfortably on the force transducer (SCAIME, ZFA, 50 kg). To collect the force data and to provide visual feedback to the participants, a customized LabView (National Instruments) program and a National Instruments acquisition card (DAQ NI-USB 6008- National Instruments) were used. The force data were sampled at 240 Hz and saved for later analysis. In front of the participants, the target area and task-specific online feedback was presented on a 19” screen. The online feedback consisted of a bar indicating the level of the produced force in% MVC in reference to the target level (see Figure 1). It was given at a sampling rate of 12 Hz (i.e., 1 every 20 points of the acquired force time-series was plotted on the screen).

Each trial was initiated by the participants starting to press the force transducer. Data collection began as soon as the average value of 20 sample points exceeded a threshold of 2 N. This was indicated to the participant by a bar next to the target area that was illuminated and remained illuminated until the trial was completed. The participants were instructed to release their force when the bar turned off. The start of each trial was self-paced, but a mandatory break of 3 s was implemented in the program. Data were stored in absolute force values.

### Tasks and procedure

Participants were informed verbally about the general procedure and were provided with written instructions explaining the task and their positioning during the trials. The experimental setup is summarized in Figure 1. Firstly, MVC was measured by using data from three trials of 5 s each. This procedure is the standard procedure to determine MVC for isometric force production with the finger [15,21,26,48,49]. The participants were instructed to press as hard as they could on the force transducer to lift then hold the mobile bar on the screen as high as possible. A resting period of about 30 s was given between trials. The mean force obtained during the last three seconds of each trial was considered as the MVC and was used to calculate the relative target force levels of the consecutive task conditions. Initial verification showed that the groups differed with regard to their MVC, *F*(1,20) = 15.65, *p* < .01, η_p_^2^ = .439 (mean ± SD, for young adults: 57.59 ± 19.40 N, and for old adults: 29.35 ± 13.58 N).

**Figure 2:**
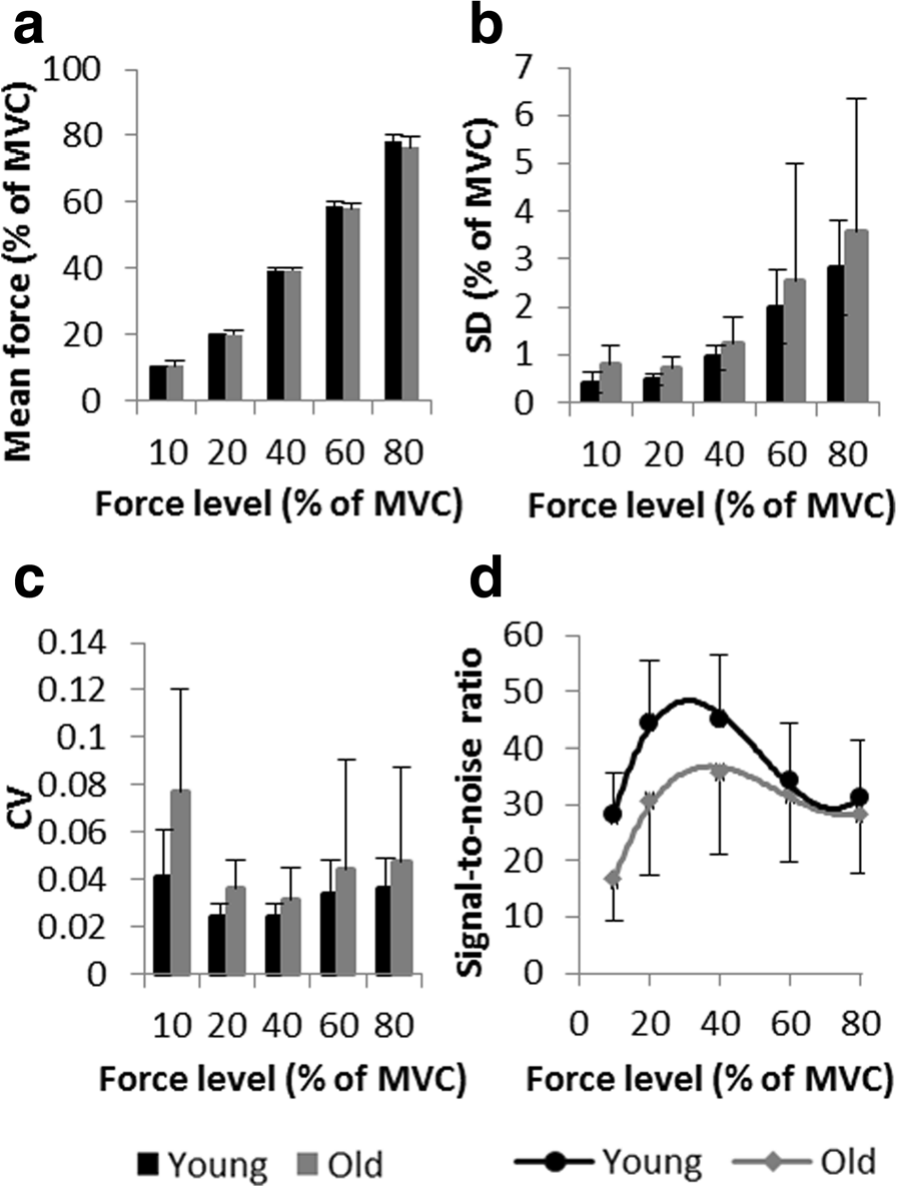
**General properties of force production.** Mean force **(a)**, standard deviation **(b)**, coefficient of variation **(c)**, and signal-to-noise ratio **(d)**, as a function of force level for young (in black) and older (in grey) participants. Each data point represents a group mean. Error bars represent the standard deviation.

Next, participants had a phase of familiarization with the experimental setup, during which they practiced all the force levels used in the following step. Afterwards, participants performed the force maintenance task at various force levels. The target force levels were 10, 20, 40, 60, and 80% of each participant’s individual MVC. The target force level to be maintained was indicated on the screen with two red lines (2 pt width), between which a blue line could be moved up and down as function of the force applied on the force transducer. The participants were instructed to align the blue line with the red ones and to maintain it as stable as possible for 15 s. Timing was indicated by the illumination of the green bar next to the display (Figure 1). Each force level condition was performed twice consecutively. The order of force levels was randomized. Verification, preliminary to data analysis, confirmed that all participants respected task instructions and that there were no significant differences between groups in terms of relative mean force production (see Figure 2a).

### Data analysis

The acquired data of the produced force over time were analyzed with the use of a Matlab R2012b customized program (MathWorks, Natick, MA, USA). The data were low-pass filtered with a 4th order Butterworth filter at 30 Hz. To exclude the ramp phase from further analysis, the first 2.5 s were discarded. All the following analyses were accordingly conducted on 12.5 s trials. Data were converted to relative force values in percentage of the participants’ individual MVC. All variables were calculated per trial and then averaged over the two trials. The mean of the produced force, the standard deviation, and the CV were calculated to characterize general properties of force production *CV = SD/mean force*. As an index of efficiency, the signal-to-noise ratio was computed as: *Signal-to-noise ratio = mean force/SD*. The variability structure of force time series was characterized using MSE as introduced by Costa and colleagues [45]. This method consists of calculating SampEn [43] over multiple time-scales contained in the analyzed time series, and thus in the expressed dynamics [11,45]. SampEn measures the degree of irregularity in the fluctuations of a time series by calculating conditional probabilities, which represent the likelihood that a vector of *m* data points matches a template with the same number of data points within a tolerance range of *r* normalized to the SD of the signal [50]. We set *r* to 0.2 and *m* to 2. The time-scales were constructed by coarse graining the original time series using a moving average with non-overlapping windows of the size (i.e., number of points) of the scale factor (see [11] for details and illustration). Then SampEn was calculated for each of the coarse grained time series. Based on our signals’ length and sampling rate, sample entropy was calculated for 60 scales. The MSE curves were obtained by plotting SampEn values as a function of the scale factor. To capture their general characteristics, based on Zhang’s definition of complexity as the integral of all scale-dependent entropies [51], the area underneath the curves was calculated (see also [52]). Then, focus was brought on the area of the time-scales range representing sensorimotor processing (i.e., containing information about 0–4 Hz bandwidth). Additionally, specific scales were examined to explore whether task and age effects on behavioral regularity show clear scale dependence that would be mediated by the control mechanisms at stake. Namely, three functionally relevant scales were selected: Scale 4 (16 ms), representative of the signals’ frequency range after filtering (i.e., 0–30 Hz); Scale 10 (41 ms), more representative of the mechanisms known to operate in force control, that is, sensorimotor processing and physiological tremor (0–12 Hz); Scale 30 (125 ms), the most representative of sensorimotor processing (0–4 Hz).

The structure of the force output in the frequency domain was characterized by spectral analysis. The power spectrum was calculated by use of the pwelch function implemented in Matlab by means of a Hanning window with 256 data points with a non-overlapping window of 1024 data points. To characterize the overall changes in the distribution of power, the slope of the log-log plot was determined per participant for each condition. It quantifies how broadband the power spectrum is, with a 0 slope being that of white Gaussian noise. Peak power was determined in the frequency band from 0–4 Hz and in the frequency band from 7–14 Hz. The window to detect peak power of the tremor component was defined after visual inspection to account for inter-individual differences in peak frequency. Additionally, proportional power spectra were calculated and the same detection of peak power within the aforementioned frequency bands was performed.

Moreover, signal-to-noise ratio and MSE areas as well as MSE values per representative scales were plotted over the force levels and polynomial fittings were performed to capture characteristics of task-related changes per group. For the MSE values per scale the fitting was used for visualization, for the signal-to-noise ratio and the MSE area the peak values per group were detected and interpreted.

### Statistics

Statistical analyses were conducted in STATISTICA (StatSoft, Tulsa, OK, USA). All dependent variables (i.e., SD, CV, signal-to-noise ratio, MSE curve areas, MSE curve areas over sensorimotor range, spectral slope, peak power and proportional peak power of the two functionally relevant frequency bands) were analyzed with an Age (2) × Force Level (5) ANOVA with repeated measures on the latter factor. In order to analyze the MSE for the specific scales, an Age (2) × Force Level (5) × Scale (3) ANOVA with repeated measures on both latter factors was calculated. The sphericity of the data was verified with the test of Mauchley. The Greenhouse-Geisser correction was applied when the epsilon value was smaller than 1 [53]. Non-adjusted degrees of freedom are reported. The level of significance was set to p < 0.05. When significant, effect sizes are given as partial Eta squares (η_p_^2^). Significant main effects and interaction effects were followed by Newman-Keuls’ post-hoc test.

## Results

### Variability of force production: SDs and CVs

Analysis of the SD (expressed in% of MVC, see Figure 2b) revealed that it increased with increasing force level, *F*(4,80) = 21.21*, p* < .01, η_p_^2^ = .401. On the other hand, no main effect of age, *F*(4,80) = 1.94*, p* = .18, and no significant age-by-force level interaction, *F*(1,20) = 0.17*, p* = .85, were observed. For the CV (see Figure 2c), statistical analysis revealed a main effect of force level, *F*(4,80) = 6.25*, p* < .01, η_p_^2^ = .238, with the relative variability in the 10% condition higher than in all other force levels (all p < .01), and a main effect of group, *F*(1,20) = 5.57*, p* = .03, η_p_^2^ = .218. Older adults were generally more variable than younger adults. The interaction of force level and age was not significant, *F*(4,80) = 1.52*, p* = .23.

### Signal-to-noise ratio and efficiency functions

The signal-to-noise ratio curves can be inspected in Figure 2d. The main effects of age, *F*(1,20) = 4.69*, p* = .04, η_p_^2^ = .190, and force levels, *F*(4,80) = 18.57*, p* < .01, η_p_^2^ = .481 were significant, along with a tendency for an age by force level interaction, *F*(4,80) = 2.56*, p* = .08, η_p_^2^ = .113. The young adults had higher values of signal-to-noise ratios than older adults for low and medium force levels (10, 20 and 40%) but not for the two higher ones. Within the group of older adults, the 10% condition was performed with a lower signal-to-noise ratio, than all other conditions, which were not different between each other (all *p* < .01). For young adults the 20% and the 40% conditions revealed higher signal-to-noise ratios than the 10%, 60%, and 80% conditions (all *p* < .01). Newman-Keuls post-hoc comparisons showed no significant pairwise age differences for each force level (i.e., 10, 20, 40, 60, and 80%). In both groups, signal-to-noise ratio curves were best fitted by a third order polynomial (both R^2^ = 0.99; note, however, that we had only five data points). These curves, representing efficiency functions, followed the same inverted-U trend for young (y = 0.0005×^3^ - 0.08×^2^ + 3.61× + 0.25) and older (y = 0.0003x^3^ - 0.05×^2^ + 2.64× - 0.25) participants. Maxima were detected for young adults at 31.5% of MVC and for older adults at 41.5% of MVC (corresponding to absolute group averages of 18.14 N and 12.18 N, respectively).

### Complexity of force output: MSE curves

#### Visual inspection

**Figure 3:**
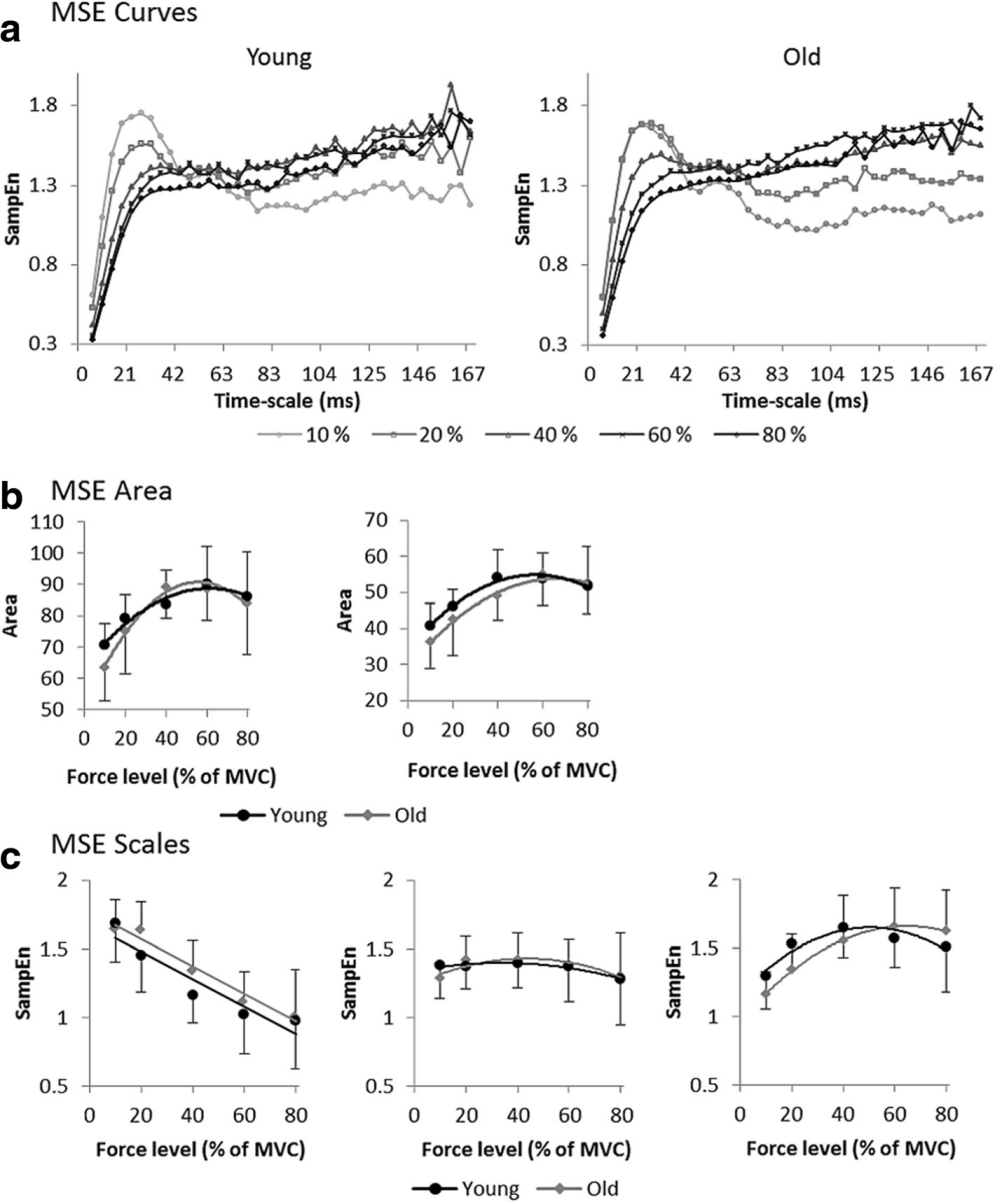
**Changes in the structure of force variability. (a)** MSE curves per force level (darker colors for higher levels) for young (left panel) and older (right panel) participants. **(b)** Mean and standard deviation of the area underneath the MSE curve for each force level. **(c)** Mean and standard deviation of SampEn values per force level for young (in black) and older (in grey) participants at scale 4 (16 ms) in the left panel, scale 10 (41 ms) in the middle panel, and scale 30 (125 ms) in the right panel.

**Figure 4:**
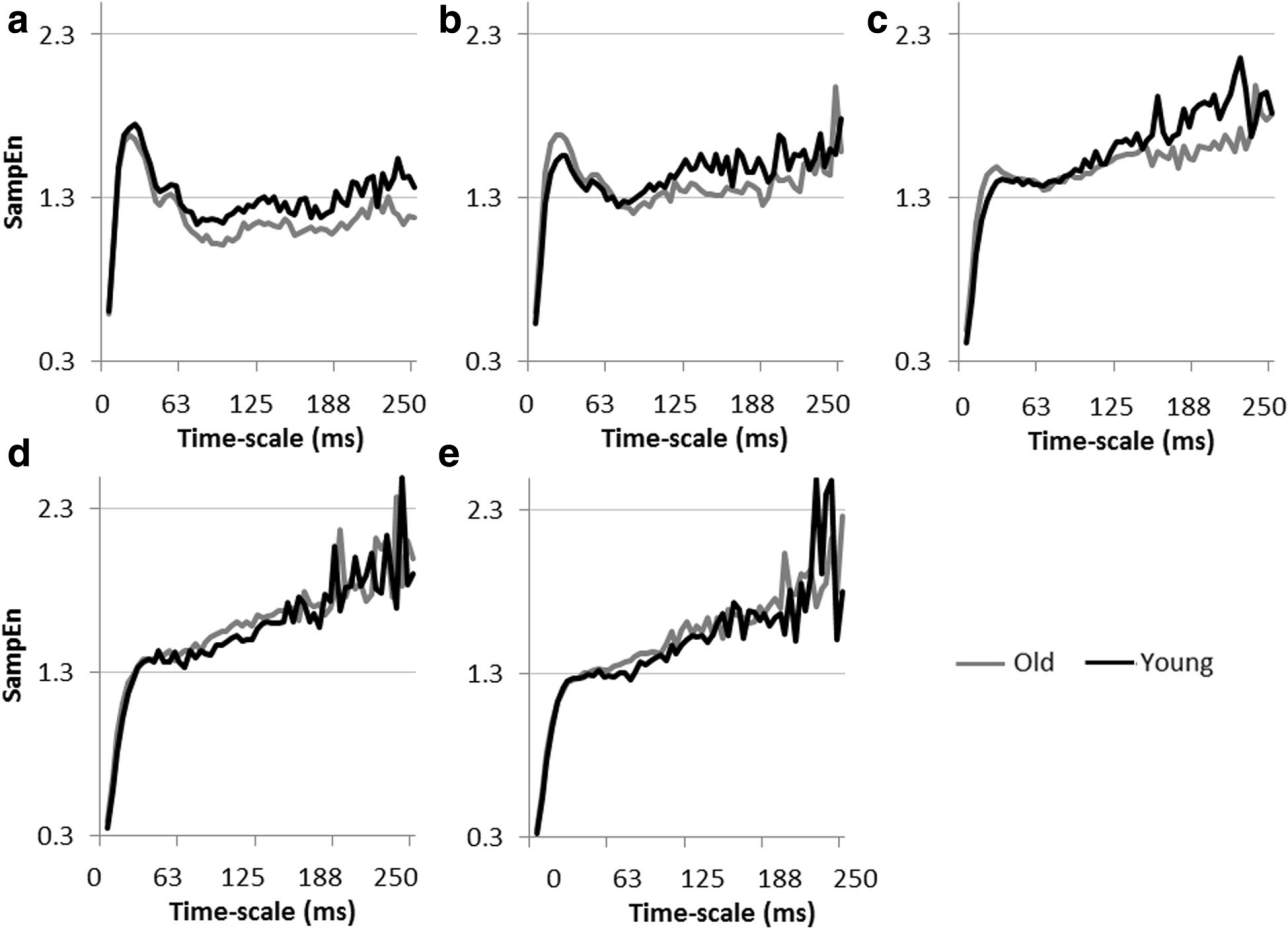
**Structure of force variability per condition.** Averaged MSE curves are plotted per group for the force levels 10 **(a)**, 20 **(b)**, 40 **(c)**, 60 **(d)**, and 80 **(e)**% of the MVC.

Figure 3a presents mean MSE curves for young and older adults at different force levels. It shows scale-dependent evolutions of SampEn with a common initial fast increase for short scales up till around scale 5 (21 ms), followed by a stabilization for longer scales (> scale 10, 42 ms). For both groups, MSE curves of the different force levels show a crossing around scale 10. Before the crossing, i.e., for shorter scales, SampEn values decreased with increasing task difficulty. Conversely, after the crossing, i.e., for longer scales, higher SampEn values were found for higher force levels. Overall, the curves suggest an increased complexity with increasing force levels. Age-related differences could be observed for low and medium force levels (10, 20, and 40%), with a consistent finding that, on average, the older participants were less entropic for most of (long) time-scales and hence showed trend for a less complex behavior (see Figure 4 for group-wise comparison of MSE curves per force level).

#### MSE curve areas

ANOVA for this comparison revealed a main effect of force levels, *F*(4,80) = 15.13*, p* < .01, η_p_^2^ = .431, but no main effect of age, *F*(1,20) = 0.98*, p* = .34, or age-by-force level interaction, *F*(4,80) = 0.84*, p* = .48. The 10% force level had the smallest area (p < .01 for all pairwise comparisons) and the 20% area was larger than the 10% area, but smaller than the 40% and the 60% areas (p < .05 for all pairwise comparisons), which were equivalent. For both young and older adults, the MSE area showed an inverted-U trend over force levels with a peak value roughly around 60% (see Figure 3-b left panel).

#### MSE curve areas range reflecting sensorimotor processing

ANOVA for this comparison showed a main effect of force levels, *F*(4,80) = 17.34*, p* < .01, η_p_^2^ = 464, but no main effect of age, *F*(1,20) = 1.98*, p* = .17, or age-by-force level interaction, *F*(4,80) = 0.85*, p* = .49. As for the total area, both young and older adults presented an inverted-U trend over force levels with a peak value roughly around 60% (see Figure 3-b right panel). Although it did not reach statistical significance at group level, older adults had, on average, lower SampEn values for the first three force levels (10 – 40%).

### Time-structure of fluctuations at functionally relevant MSE scales

A Group (2) × Scale (3) × Force level (5) analysis of variance revealed: (i) main effects of force level, *F*(4,80) = 3.47*, p* = .02, η_p_^2^ = .148, and scales, *F*(2,40) = 12.21*, p* < .01, η_p_^2^ = .379, (ii) a scale-by-force level interaction, *F*(8,160) = 50.11*, p* < .01, η_p_^2^ = .715, and (iii) a significant three-way interaction, *F*(8,160) = 1.91*, p* = .02, η_p_^2^ = .108, statistically showing the described observations of time-scale dependent entropy changes. Main effect of age, *F*(1,20) = 0.09*, p* = .77, and the interactions of age-by-scale, *F*(2,40) = 1.65*, p* = .21, and age-by-force level, *F*(4,80) = 0.70*, p* = .55, were not significant. Post-hoc comparisons revealed differential results for each scale. For scale 4, in young adults, SampEn was the highest at the 10% condition. In addition, the SampEn observed at the 20% condition was higher than the other three force levels (all *p* < .05). At the 40% condition, older adults yielded lower complexity values than at the 10 and 20% conditions (both *p* < .01), but higher values than at the 80% condition (*p* = .03) and marginally higher values than at the 60% condition (*p* = .07). For scale 10, no differences between force levels were revealed in young adults. Similar to the group of young adults, no significant differences between force levels were observed in older participants. For scale 30, in young adults, the 40% condition had higher SampEn values than the 10% condition (*p* = .01). In older adults, SampEn at 40, 60, and 80% was higher than at 10%, and SampEn for 60% was higher than those observed for 20% (all *p*’s < .05). In line with post-hoc results, looking at the entropy values for the different force levels per scales revealed different patterns: for scale 4, the entropy decreased with increasing force level (Figure 3c left panel); for scale 10, the entropy curve was almost flat (Figure 3c middle panel); and for scale 30 (Figure 3c right panel), a marked inverted-U shape was observed, closely resembling the signal-to-noise ratio curves, with a peak around 40% for young adults (2^nd^ order fit: y = −0.01×^2^ + 1.41× + 51.19; R^2^ = 0.98) and at 60% for older participants (2^nd^ order fit: y = −0.07×^2^ + 0.81× + 63.80; R^2^ = 0.95). Although it did not reach statistical significance at group level, at the short time-scale, which reflects both fast and slow dynamics, older adults had, on average, more entropic signals (i.e., less regular) than younger adults for intermediate force levels. Conversely, when looking at the longer time-scale, older adults expressed more regularity for the first three force levels (cf. Figure 3c right panel).

### Frequency-structure of force output

**Figure 5:**
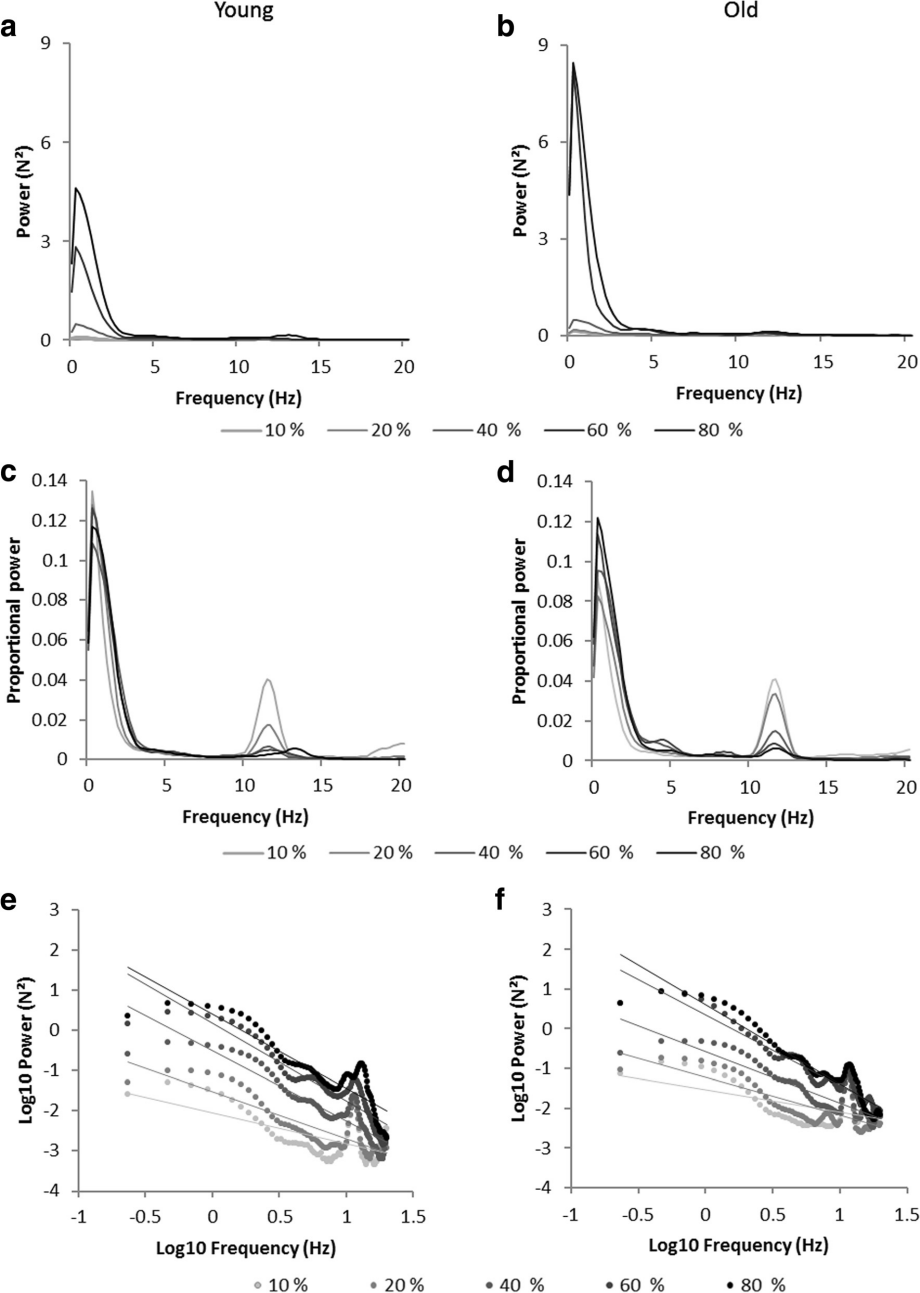
**Characterization of frequency spectra.** Curves represent group mean values for young (left) and older (right) adults per force level. **(a)** and **(b)** Power spectrum for frequencies between 0 and 20 Hz (representing approximately 95% of the total power for each force level). **(c)** and **(d)** Proportional power spectrum for frequencies between 0 and 20 Hz. **(e)** and **(f)** Log10 power as a function of the log10 frequency for each force level with spectral slopes determined by linear regression (note: for data analysis slopes are calculated based on individuals frequency spectra).

**Figure 6:**
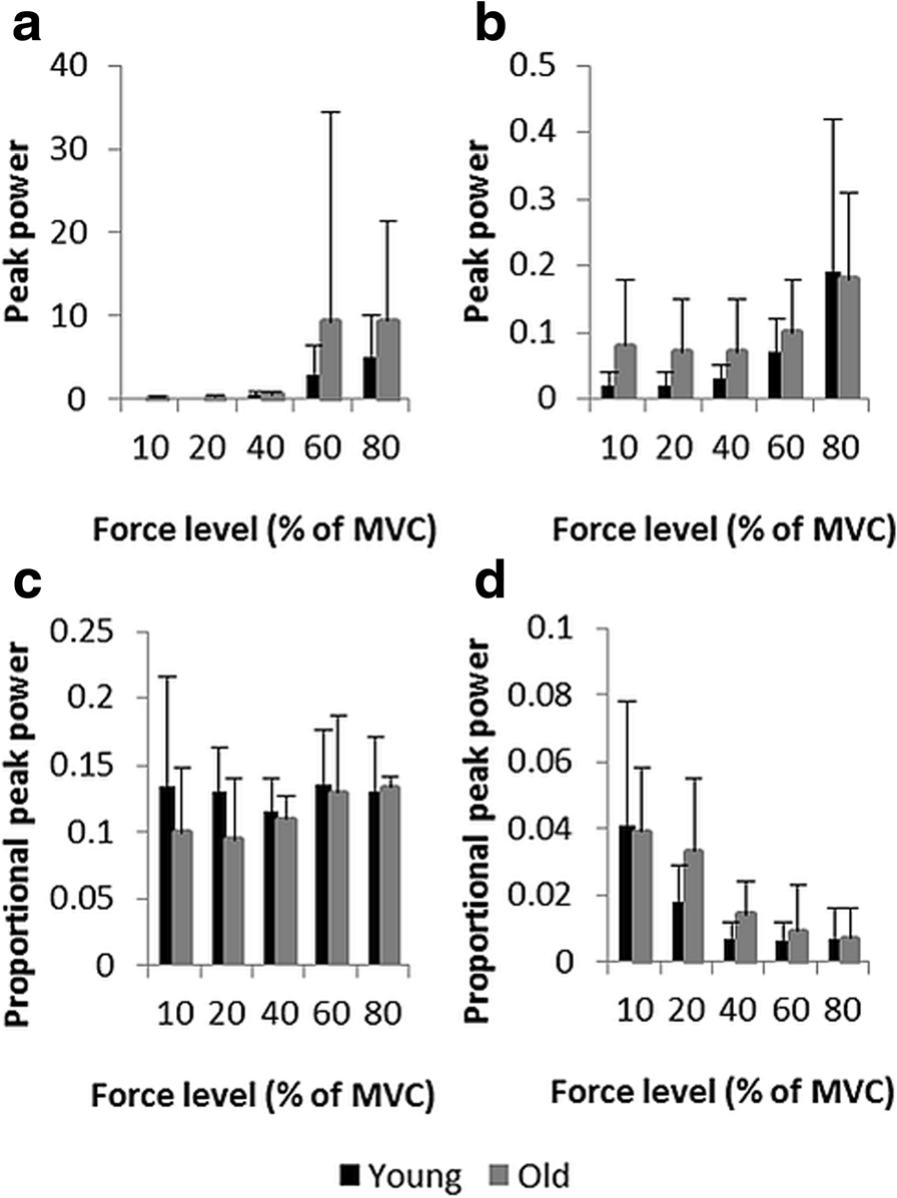
**Peak power per force level in the functionally relevant frequency bands. (a)** and **(b)** Mean and standard deviation of peak power of the frequency bands reflecting sensorimotor processing and tremor respectively, with their corresponding proportional peak power values in **(c)** and **(d)**.

Absolute and proportional frequency spectra and log-log frequency-power plots per group are presented in Figure 5. Peak power and proportional peak power are plotted in Figure 6 as a function of force level.

### Spectral slope

The spectral slope differed between force levels, *F*(4,80) = 51.05*, p* < .01, η_p_^2^ = .720. The values of the slopes increased with increasing force levels (see Table 1) indicating a decrease in the spread of power across the spectrum with increasing task demands. The slopes observed in the 10% and 20% conditions (−0.95 and −1.21) were significantly lower than the three other force levels (40% = −1.64, 60% = −1.78 and 80% = −1.87, respectively). The slope observed in the 40% condition was lower than those observed for the 80% condition (p < .01). Main effect of age, *F*(1,20) = 2.87*, p* = .11, and age-by-force level interaction, *F*(4,80) = 0.69*, p* = .49, were not significant.

**Table 1:** **General properties of force production**

**Force level**	**10%**		**20%**		**40%**		**60%**		**80%**	
**Group**	**Young**	**Old**	**Young**	**Old**	**Young**	**Old**	**Young**	**Old**	**Young**	**Old**
**Measure**	**Mean (SD)**	**Mean (SD)**	**Mean (SD)**	**Mean (SD)**	**Mean (SD)**	**Mean (SD)**	**Mean (SD)**	**Mean (SD)**	**Mean (SD)**	**Mean (SD)**
Mean	10.06 (0.34)	10.45 (1.64)	19.78 (0.44)	19.92 (1.14)	39.26 (0.80)	39.33 (1.11)	58.54 (1.62)	57.94 (1.81)	78.05 (2.17)	76.50 (3.45)
SD	0.41 (0.22)	0.79 (0.40)	0.47 (0.11)	0.71 (0.24)	0.94 (0.25)	1.22 (0.58)	1.99 (0.77)	2.52 (2.49)	2.82 (0.98)	3.56 (2.78)
CV	0.04 (0.02)	0.08 (0.04)	0.02 (0.01)	0.04 (0.01)	0.02 (0.01)	0.03 (0.01)	0.03 (0.01)	0.04 (0.05)	0.04 (0.01)	0.05 (0.04)
Index of efficiency										
S-t-N	28.14 (7.35)	16.53 (7.36)	44.53 (11.09)	30.67 (13.16)	45.13 (11.42)	35.71 (14.49)	34.08 (10.19)	31.86 (12.17)	31.09 (10.35)	28.34 (10.67)
Frequency analysis										
Slope	−1.02 (0.33)	−0.88 (0.49)	−1.31 (0.35)	−1.05 (0.31)	−1.74 (0.32)	−1.55 (0.28)	−1.92 (0.26)	−1.65 (0.36)	−1.89 (0.32)	−1.85 (0.38)
Peak power (0–4 Hz)	0.05 (0.03)	0.15 (0.12)	0.10 (0.03)	0.21 (0.20)	0.52 (0.41)	0.54 (0.27)	3.00 (3.36)	9.23 (25.18)	5.14 (4.83)	9.35 (11.99)
Prop power (0–4 Hz)	0.134 (0.082)	0.099 (0.049)	0.130 (0.033)	0.094 (0.046)	0.115 (0.025)	0.109 (0.018)	0.136 (0.040)	0.129 (0.058)	0.130 (0.041)	0.133 (0.009)
Peak power (7–15 Hz)	0.02 (0.02)	0.08 (0.10)	0.02 (0.02)	0.07 (0.08)	0.03 (0.02)	0.07 (0.08)	0.07 (0.05)	0.10 (0.08)	0.19 (0.23)	0.18 (0.13)
Prop power (7–15 Hz)	0.041 (0.038)	0.039 (0.019)	0.018 (0.011)	0.033 (0.022)	0.007 (0.005)	0.014 (0.010)	0.006 (0.006)	0.009 (0.014)	0.007 (0.009)	0.007 (0.009)
Multiscale complexity analysis (SampEn)										
Area	70.53 (6.75)	63.32 (10.43)	79.18 (7.59)	74.96 (13.59)	88.98 (9.94)	83.42 (10.95)	88.29 (9.94)	90.24 (11.65)	84.10 (16.40)	85.87 (14.39)
Area 30-60	40.83 (6.24)	35.98 (7.01)	46.13 (4.89)	42.41 (9.89)	54.08 (7.71)	48.87 (6.54)	53.92 (7.08)	54.68 (8.09)	51.86 (10.95)	52.44 (8.22)
Scale 4	1.69 (0.21)	1.64 (0.29)	1.45 (0.20)	1.63 (0.26)	1.16 (0.21)	1.34 (0.21)	1.02 (0.22)	1.12 (0.29)	0.97 (0.34)	1.01 (0.35)
Scale 10	1.38 (0.11)	1.29 (0.23)	1.38 (0.17)	1.42 (0.17)	1.40 (0.19)	1.42 (0.18)	1.37 (0.18)	1.38 (0.26)	1.28 (0.31)	1.30 (0.34)
Scale 30	1.29 (0.17)	1.16 (0.24)	1.53 (0.26)	1.34 (0.19)	1.65 (0.33)	1.55 (0.22)	1.57 (0.28)	1.65 (0.21)	1.51 (0.30)	1.62 (0.33)

Means and standard deviations of the general properties of force production, signal-to-noise ratio (S-t-N), as well as results of the frequency and MSE analyses.

### Peak power of the functionally relevant frequency bands

#### Sensorimotor processing band

For peak power, in the frequency range of 0–4 Hz, analysis revealed a tendency toward a main effect of force level, *F*(4,80) = 3.37*, p* = .06, η_p_^2^ = .144. Overall, the power increased with force level. Post-hoc comparisons showed that the peak power observed at 80% was higher than those observed at 10%, 20%, and 40%. In addition, peak power observed in the 60% condition was higher than that observed at the 40% condition. Main effect of age, *F*(1,20) = 1.60*, p* = .22, and age-by-force level interaction, *F*(4,80) = 0.83*, p* = .52, were not significant. However, older participants had, on average, greater absolute peak power than younger adults at the two high force levels (see Figure 5a, b for peaks of the mean curves and Figure 6a for mean peak power values). Analysis of proportional power revealed no significant effect of age, *F*(1,20) = 1.89*, p* = .18, force level, *F*(4,80) = 1.34*, p* = .27, and no significant interaction, *F*(4,80) = 0.97*, p* = .42. Nevertheless, mean curves indicated a higher proportional power for older adults for the lowest force levels and an increase in proportional peak power with force level for the group of young adults (see Figure 5c, d for the mean curves and Figure 6c for mean peak power values across force levels).

#### Physiological tremor band

The amount of peak power in the tremor component showed a main effect of force level, *F*(4,80) = 9.40*, p* < .01, η_p_^2^ = .320, being the highest for 80% (*p* < .01). The age effect, *F*(1,20) = 3.21*, p* = .09, η_p_^2^ = .138, and the age-by-force level interaction were not significant, *F*(4,80) = 0.32*, p* = .40, although older adults displayed, on average, greater peaks for low and medium force levels (see Figure 5a, b for the mean curves and Figure 6b for mean peak power values). The analysis of proportional power showed no main effect of age, *F*(1,20) = 1.18*, p* = .29, and the age by force level interaction, *F*(4,80) = 1.01*, p* = .36, failed to reach significance. The proportional peak power differed for the force levels, *F*(4,80) = 19.85*, p* < .01, η_p_^2^ = .498. It decreased as task requirements increased (see Figure 5c, d for the mean curves and Figure 6d for mean peak power values). At 10% highest proportional peak power was revealed and proportional peak power at 20% was higher than at 40, 60, and 80% (all *p* < .001).

## Discussion

In the present study, we investigated the sensorimotor processes underlying isometric force control through measures of the magnitude and the time-frequency structures of behavioral variability.

As a preliminary observation, our participants groups presented significant differences in MVC, with older participants’ values being lower than those of younger ones. This result was expected and can be considered as a typical marker of age-related dynapenia [30,31]. It is noticeable however that the observed group difference in MVC is considerably larger than what is usually reported in the literature (see for example [18,40,54], and [25,39] where no differences were found), which insured that we tested here significantly different force control systems. However, the use of relative force levels, which is a procedure commonly used in the literature to normalize for inter-individual strength disparity [15,21,34,37,40,55], also insured that force conditions were comparable between participants. In this respect, we found that participants of both groups were equally capable of correctly scaling their produced force and performing the task according to the instructions.

### Variability and efficiency differ between force levels in young and older adults

Absolute variability of force outputs (SD) non-linearly increased with force level in both groups. Relative variability (CV) was higher at the lowest force level (10%) than for all the other levels and older adults were more variable than young participants. These results are consistent with previous studies reporting that CV is an age-sensitive measure [56-58] and that the age effect is more pronounced for relative than for absolute variability [39,59]. They are also consistent with Christou’s [60] assumption that age effects on force output variability are more consistently observed for lower than for higher force levels. Overall, age-related changes in variability can also be related to those reported using other tasks. Indeed, the observed increase in relative variability of force output with age (CV) is consistent with the general observation that older adults are more variable than young adults in a wide range of cognitive (see [61] for an overview) and motor tasks, e.g., repetitive reaching [62] or gait [63].

The efficiency of information processing of the force control system was estimated through the use of the signal-to-noise ratio (see [15] for a similar procedure), which showed an inverted-U shape in both groups, with a peak value around 35% of the MVC in young adults, and around 40% in older adults. This result is consistent with the findings reported by Slifkin and Newell [15,34] in young adults. It shows additionally that, while conserving a general inverted-U shape in both groups, aging does not dramatically affect the range of optimal functioning relative to the maximum force level. Results also showed a lower signal-to-noise ratio in older adults, which supports the hypothesis that aging leads to a decrease in information processing efficiency in the central nervous system. This has been already observed in both the sensorimotor (e.g., [9,6]) and the cognitive domains (e.g., [64,65]). Nonetheless, the difference between young and older adults was significant at low and medium force levels, with the largest difference at the level corresponding to optimal signal-to-noise ratio (i.e., optimal information transmission) and no difference was found for the higher force levels. Although the maximization of age differences for intermediate relative force levels is currently admitted for force control (see [66]), such pattern differs from that observed with different paradigms, as for instance in Fitts’ task [66] and, more generally, from the *age-by-complexity effect* [67,68], which has been reported in aging literature. Indeed, the relation between task difficulty and performance is, in general, described as being linear and age-related differences as being more pronounced with increasing task difficulty. Presumably, the present different pattern of age-by-complexity effect reflects the specificities of the task (see [69] for a comparable argumentation). Indeed, in isometric force control tasks, the difficulty does not seem to increase linearly with force level, as attested by the inverted-U shape of the signal-to-noise ratio in both groups. However, the shapes of the efficiency functions are slightly different between the two groups, i.e., more marked in young participants, thereby leading to a specific pattern of age differences. The observed age-related differences presumably also depend on whether absolute or relative variability is measured. As an illustration, we did an exploratory analysis by studying the force levels for which younger and older participants had on average comparable absolute force levels. By doing so, group differences in variability (SD) were reversed such as higher differences were observed for the higher force levels. Similarly, older participants were found to have lower signal-to-noise ratios at higher force levels (12 and 24 N, 20% and 40% for young, 40 and 80% for older participants, p < .05), but were equivalent at the low force level (6 N, 10% for young, 20% for older participants, p > 0.05).

### Frequency structure of force fluctuations across force levels in young and older adults reflects different sensorimotor processing components

The frequency structure of the force output fluctuations was captured by the power distributions of frequency spectra and by the spectral slopes. With respect to the power spectra analysis, results showed that the power distribution changed comparably in both groups as a function of force level. Specifically, the spectral power distribution showed two peaks that were located in the frequency ranges presumably representing sensorimotor processing and tremor, respectively. This distribution structure is consistent with the findings previously reported by Taylor et al. [70], whereas it remained unobservable in other studies that focused on the frequency range representing sensorimotor processing [15,34].

Overall, for both frequency bands absolute peak power increased with force levels. This effect was previously reported for the frequencies reflecting sensorimotor processing (e.g., [15]). Proportional power analysis showed that, compared to absolute power, the effect of force level was diminished in the frequency range reflecting sensorimotor processing, and reversed for the tremor component (i.e., the highest peak power observed for the lowest force levels). These results suggest that force levels change the relative contribution of the sensorimotor processing and the tremor components to the produced behavior, which is reflected in different frequency structures of force fluctuations. Such changes are accompanied by changes toward steeper spectral slopes with increasing force level. This indicates that the frequency content of force output is not randomly distributed, but rather dominated by structured fluctuations. The contribution of lower frequencies increases as the relative force requirements increase.

It is striking that similar frequency profiles and spectral slopes were observed for both age groups. These results differ from those observed in previous, but not strictly comparable, studies where higher absolute power values were reported for older than for younger participants, for both frequency ranges, i.e., 0–4 Hz [37] and 8–12 Hz [71,72], respectively. With regard to proportional power analysis, current literature is not unanimous when it comes to age effects. For instance, looking at age-related differences in low relative force production (15% MVC), Sosnoff and Voudri [73] found higher proportional power for older participants than for younger ones, but not for the frequencies representing sensorimotor processing. Conversely, Heffernan et al. [66], who used the 30% MVC force condition, for which the highest group difference was expected, did not report a significant age effect on any of the examined frequency ranges (namely, 0–4, 4–8, and 8–12 Hz). Other studies also did not report age-related differences for the frequency range related to physiological tremor [2,74,75]. In this respect, Morrison and Newell [32] proposed two possible explanations: (i) the increase of tremor might be only detectable for participants older than 80 years; and (ii) the task conditions under which the tremor is assessed might be decisive, with more challenging situations (e.g., involving multiple segments) being more affected by age (see also [76]). However, as we didn’t study tremor explicitly (i.e., we only captured the tremor component in force control), we refrain from drawing extensive and speculative interpretations. At least, it might be that, for both the sensorimotor and tremor components, discrepancies between the present results and those reported in previous studies result from group differences in absolute force levels.

In addition to the analysis of frequency structure of fluctuations, we also investigated variability structure in the time domain.

### Complexity and time-scale dependent structure of force output change across force levels in young and older adults

The changes in the time structure of force output fluctuations were captured by the MSE analysis. To our knowledge, no previous study has investigated age-related differences of MSE of force output fluctuations across a wide range of relative force levels. This metric is considered as a more reliable estimator of the system’s complexity than single scale measure of entropy, e.g., ApEn and SampEn. Indeed, restricting the analysis to the original time-scale of the acquired time-series conveys simply information about its regularity, which could change depending on the used sampling frequency and filtering (e.g., [11]). Thus, plotting MSE curves provided information about the richness of the signal across the different scales, as a function of increasing force levels and age.

Results showed that, across groups and force conditions, the overall shape of MSE curves was prominently characterized by an eventual stabilization, or a slight increase, of SampEn values over longer time-scales. The observed MSE profiles clearly differ from those corresponding to random signals, e.g., white noise, for which entropy values dramatically decrease beyond the first scales (cf., [11]). Instead, they indicate the presence of long-range correlations that are not dominated by a single time constant. The presence of these non-random fluctuations of force outputs reflects the inherent complexity of the force control system, which implicates the interaction of somatosensory and visual feedback networks, numerous brain regions, and muscular processes operating over multiple time scales. In addition, these long-range correlations constitute non-invasive markers to elucidate multi-scale contribution of specific task- or system-related constraints.

For both age groups, the increase in force level resulted in an increase in SampEn values for most of the higher scales, thereby suggesting an increase in long-term correlations. For each force level, a visual inspection of group mean curves suggested lower complexity in the elderly for the low and intermediate force levels (<60%), as shown by the lower entropy at most longer scales (>10; 42 ms). The whole area underneath the MSE curve plotted as a function of force reveals an inverted-U shape for both groups. This pattern indicates an optimal complexity around 60% of MVC, which is a higher percentage than those corresponding to the optimum of the signal-to-noise ratio in young and older participants groups. The area underneath the scales representing the sensorimotor processing component (scale 30–60; 125 ms - 250 ms) also displayed a similar pattern of results. It is noticeable, however, that, although the visual inspection of MSE curves suggested a decrease in complexity with increasing age, this was not confirmed by the statistical analysis. This result does not allow us to draw conclusions consistent with the currently admitted loss of complexity hypothesis (LOCH, [13]), which predicts a decrease in behavioral complexity with aging (for reviews see [9,10]). The reasons that explain such lack of statistical evidence are not completely clear. However, it can be noticed that most of previous studies, including those on force control, inferring or predicting age-related changes in complexity in support of the LOCH have used single scale entropy measures (e.g., see [13] or [8,10] for reviews).

Though a single scale cannot be used to faithfully describe the overall dynamics, which emerges from interactions between processes operating at multiple time-scales, we contend that it could be of interest to assess specific control processes. This was supported by the three-way interaction of age, force level, and scale, which revealed a differentiated pattern of the signal’s regularity at time scales related to the different components of force output fluctuations. For instance, the analysis of scale 4 (16 ms), which characterizes the overall dominant/meaningful fluctuations of the force output, revealed that behavioral regularity increased (i.e., SampEn decreased) with increasing force level. This result corroborates the findings of the spectral slope analysis, and supports the hypothesis that with increasing force level more long-term correlations are present. Conversely, for scale 10 (41 ms) including frequencies up to 12 Hz (tremor frequency) and around the crossing of MSE curves of different force levels, no effect of task conditions was observed on SampEn values. It can be concluded that, after high frequencies were averaged out from the signal (following the coarse graining procedure), the structure of force fluctuations was found to be similar across all force levels. At scale 30 the sensorimotor processing component can be more specifically examined. Here, as expected, results showed an inverted-U shape pattern that is comparable to the MSE area and to the signal-to-noise ratio curves. Although no clear pattern of age differences could be extracted, the observations related to the interaction of group factor with force level and time-scale suggest that, compared to young adults and depending on the control processes under scrutiny, elderly could show more or less structured behavior at different force levels.

Overall, on the one hand, our results show that young and elderly participants adopt a qualitatively similar organization of the force control system that is reflected by the time-structure of fluctuations and the similar changes in response to task requirements. However, it is noticeable that these findings were highlighted by confronting young and elderly to comparable constraints that is, by controlling for the age-related deficit in strength through the use of relative force. On the other hand, group differences did not completely vanish, which suggests that age differences can be more or less maximized, depending on task settings. For instance, if task difficulty was scaled in absolute instead of relative values, or if different degrees of freedom or muscular synergies were used, age effects could be presumably larger (for converging findings, see [77], where different structures of variability are reported when generating isometric forces in two different directions). Thus, the present observations suggest that, at least in isometric force control tasks, a coalition of multiple factors determines how the system organizes its degrees of freedom to accomplish the task, which is reflected in the amplitude and structure of variability of behavioural outputs. This perspective is consistent with Morrison and Newell’s [32] assumption that the confluence of constraints including aging, instead of the aging process itself, determines the complexity of force outputs. Further research is still needed to disentangle the contributing processes to force control and their interactions at various task settings and in different groups.

### Correspondence between efficiency, complexity, and frequency structure observed in young and older adults

The use of several measures provides multiple markers to characterize variability, complexity, and efficiency of the force control system and to capture information about the contribution of the underlying sensorimotor processes. Therefore, considering these measures in conjunction, instead of separately, might afford a global picture of how the force control system is organized in principled ways under different task conditions and whether this organization changes during aging.

Inspired by Slifkin and Newell’s [15] study, we searched for the correspondence between the evolutions of signal-to-noise ratio and complexity measure (i.e., the area underneath the MSE curve), as well as entropy at specific scales, as function of force levels. This was theoretically grounded on the presumed functional relation between information processing efficiency and systemic organization [42]. We extended this reasoning to the force control system under the hypothesis that such a correspondence would indicate that an optimal information transmission between the different subsystems results from an optimal organization of the underlying physiological connectivity. However, in the present study, optima of the system’s efficiency (i.e., signal-to-noise ratio), and its complexity were not aligned. In addition, since the proportional peak power showed no differentiated pattern between force levels, we could not confirm that the peak power mirrors the function of the information transmission measure. Thus, our results were consistent with those previously reported by Slifkin and Newell [15,34] with respect to the profiles of the efficiency and complexity patterns over the range of force levels, but not in terms of their correspondence. To avoid misleading conclusions, it is noteworthy to acknowledge that the present study is not directly comparable to those of Slifkin and Newell [15,34] that use ApEn, which refers to regularity and is therefore considered as insufficient to assess the changes in complexity [11]. Moreover, unlike MSE, their single scale measure doesn’t offer the possibility of focusing on multiple specific and functionally-relevant time-scales. Indeed, in the present study, the adopted multiscale approach revealed the presence of strong time-scale dependence in the observed changes in behavioral regularity. This finding implies different involvements of the control mechanisms in the structure of force fluctuations at the different force levels. In particular, it is noticeable that the inverted-U profiles of complexity curves were only observed, in both age groups, at scale 30, which is the scale capturing closely the functioning of sensorimotor processes. The convergence between MSE and power spectra shows how the increase in force level modifies the contribution of the two control processes. More specifically, it reflects the decrease in the relative implication of the tremor component with increasing force requirements. In the frequency domain, this was shown by the decrease of the proportional peak power of the 7–14 Hz range, as well as by the steeper spectral slope with increasing force level. In the time domain, it can be seen by the change in the overall shape of the MSE curves. In particular, the presence of a peak at short scales [78] for the curves at the lowest two force levels indicates that some meaningful information is contained at higher frequencies (i.e., within the tremor range). Conversely, the flatter profiles observed for 40–80% conditions indicate that, under these task constraints, the signal’s entropy is rather uniformly and predominantly contained in lower frequencies, i.e., within the sensorimotor range, which underlies the prominence of long-range correlations in force fluctuations.

## Conclusions

In the present study, we used MSE to assess changes in long-range correlations in force output fluctuations and to make the link with the findings observed with other conventional analyses (i.e., SD, CV, signal-to-noise ratio, frequency spectra and spectral slope). As currently argued within the framework of dynamical system analysis [78-80], our results support the view that behavioral variability, in terms of both magnitude and structure, have a functional meaning and provide non-invasive markers of the adaptations of the whole force control system to various constraints. Specifically, the time and frequency structures of force outputs can be used to assess the dominance of processes underlying force control. Here, we found that, for both age groups task adaptation presented a strong dependence on time-scales, with force control being increasingly dominated by long-scales (low frequencies) dynamics as force requirements increased. Although not statistically significant, a tendency for an age-related loss of complexity was observable for the easy and moderate force levels. The surprisingly weak age effects on variability and complexity measures suggests a similar organization of the system underlying force control for participants of both age groups, even though efficiency was lower in older as compared to younger participants. Nevertheless, we argue that the use of relative scaling could have attenuated age effects. Indeed, it could be that by normalizing for the most prominent age-related deficit, which in our task is force weakness, we masked age-related changes in the dynamics. For instance, to reveal how aging modifies the spontaneous dynamics in the widely used bimanual coordination paradigm, task constraints are commonly scaled in absolute terms (see [81]).

Overall, our findings suggest that the behavioral expression of the LOCH is not as straightforward as conventionally admitted (see [10] for a converging point of view). Accordingly, a number of results reported in the literature should be interpreted with caution.

## Abbreviations

NMSS: Neuro-musculo-skeletal system
ApEn: Approximate entropy
MVC: Maximal voluntary contraction
SD: Standard deviation
CV: Coefficient of variation
SampEn: Sample entropy
MSE: Multi-scale entropy
LOCH: Loss of complexity hypothesis
